# Treatment of breast fibroadenoma with high intensity focused ultrasound (HIFU): a feasibility study

**DOI:** 10.1186/2050-5736-3-S1-O78

**Published:** 2015-06-30

**Authors:** David Brenin

**Affiliations:** 1 University of Virginia, Charlottesville, Virginia, United States

## Background/introduction

Fibroadenomas are common, benign lesions of the breast. A minority of fibroadenomas will disappear without treatment, but most increase in size or remain unchanged. Current management of patients with fibroadenomas in the United States varies on a case by case basis and includes observation or surgical excision. Many patients find their fibroadenoma bothersome, and opt for surgical excision. The objectives of this study are to evaluate the safety and feasibility of Ultrasound guided High Intensity Focused Ultrasound (USgHIFU) delivered by the Echopulse device (Theraclion, Paris) for treatment of breast fibroadenomas. General patient safety, cosmetic outcome, tumor response, patient experience, physician/operator experience, and device performance will be assessed.

## Methods

Twenty female patients diagnosed with palpable, non-calcified breast fibroadenomas 1cm or larger will be enrolled in a single arm clinical trial and undergo treatment of their tumor utilizing a computer-driven, continuously cooled, extra-corporal HIFU probe mounted on an arm moved by motors, and guided in real-time with an integrated ultrasound imaging scanner. The integrated probe is positioned by the operator and the lesion is imaged. Treatment planning is automated and presented for review and approval on an integrated computer screen. Optimal energy per sonication is established for each patient by determining the minimal setting found to produce bubbles within the lesion as observed on real-time B-mode ultrasound. Patients will have tumors meeting the following criteria: Distance from the skin of ≤ 23 mm to the posterior border of the fibroadenoma, ≥ 5 mm from the anterior border of the fibroadenoma, and ≥ 11mm from the focal point of the HIFU treatment. The chest wall must be more than 1cm from the posterior margin of the tumor, and tumor volume must be between 0.3cc and 10cc.

## Results and conclusions

Subjects will be evaluated immediately after treatment and at 3, 6, and 12 months. Primary endpoints assessed will include:

• Palpability of breast lesion at 12 months following HIFU treatment session

• Patient-rated pain of the HIFU treatment assessed after completion of the treatment session

• Patient satisfaction at 3, 6 and 12 months following HIFU treatment session

• Change in volume of the fibroadenoma compared to baseline at 3, 6 and 12 months after the IFU session as assessed by ultrasound

Secondary endpoints assessed will include:

• Palpability of breast lesion at 3 and 6 months following HIFU treatment session

• Cosmetic evaluation at 3, 6 and 12 months following HIFU treatment session

• Investigator-rated evaluation of the device

• Incidence of local and/or general adverse events and other associated symptoms at 3, 6 and 12 months follow-up

• Treatment parameters including duration of the treatment session and device energy settings

• The IDE application for this study has been approved, and the trial is under accrual.

**Figure 1:**
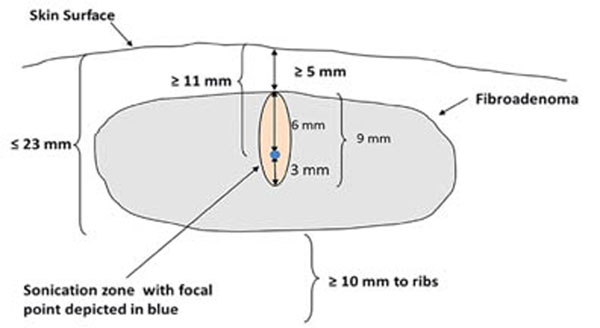
Minimum Tumor Criteria

